# Calcium Application Enhances Drought Stress Tolerance in Sugar Beet and Promotes Plant Biomass and Beetroot Sucrose Concentration

**DOI:** 10.3390/ijms20153777

**Published:** 2019-08-02

**Authors:** Seyed Abdollah Hosseini, Elise Réthoré, Sylvain Pluchon, Nusrat Ali, Bastien Billiot, Jean-Claude Yvin

**Affiliations:** Plant Nutrition Department, Centre Mondial de l’Innovation Roullier, 35400 Saint Malo, France

**Keywords:** oxidative stress, metabolites, magnesium and silicon nutrition, carbohydrate synthesis

## Abstract

Numerous studies have demonstrated the potential of sugar beet to lose the final sugar yield under water limiting regime. Ample evidences have revealed the important role of mineral nutrition in increasing plant tolerance to abiotic stresses. Despite the vital role of calcium (Ca^2+^) in plant growth and development, as well as in stress responses as an intracellular messenger, its role in alleviating drought stress in sugar beet has been rarely addressed. Here, an attempt was undertaken to investigate whether, and to what extent, foliar application of Ca^2+^ confers drought stress tolerance in sugar beet plants exposed to drought stress. To achieve this goal, sugar beet plants, which were grown in a high throughput phenotyping platform, were sprayed with Ca^2+^ and submitted to drought stress. The results showed that foliar application of Ca^2+^ increased the level of magnesium and silicon in the leaves, promoted plant growth, height, and leaf coverage area as well as chlorophyll level. Ca^2+^, in turn, increased the carbohydrate levels in leaves under drought condition and regulated transcriptionally the genes involved in sucrose transport (*BvSUC3* and *BvTST3*). Subsequently, Ca^2+^ enhanced the root biomass and simultaneously led to induction of root (*BvSUC3* and *BvTST1*) sucrose transporters which eventually supported the loading of more sucrose into beetroot under drought stress. Metabolite analysis revealed that the beneficial effect of Ca^2+^ in tolerance to drought induced-oxidative stress is most likely mediated by higher glutathione pools, increased levels of free polyamine putrescine (Put), and lower levels of amino acid gamma-aminobutyric acid (GABA). Taken together, this work demonstrates that foliar application of Ca^2+^ is a promising fertilization strategy to improve mineral nutrition efficiency, sugar metabolism, redox state, and thus, drought stress tolerance.

## 1. Introduction

Sugar beet (*Beta vulgaris* L.) is an industrial crop which belongs to the Chenopodiaceae family [1]. It provides nearly one-fifth of the sugar consumed in the world and it is also used as a significant source for the production of ethanol as bioenergy [1]. In sugar beet, sucrose comprises up to 18% of plant fresh weight which is the major form of reduced carbon involved in long-distance transport in most crop plants [2]. During photosynthesis, starch is synthesized and stored in the chloroplast matrix and sucrose is synthesized in the leaf cytosol, and through phloem transport is allocated to sink tissues where it can accumulate to high concentrations [3].

Drought is one of the major limiting factors for crop production. The reduction of sugar beet yield has been frequently reported under water limiting conditions [4,5]. The potential reduction of 5 to 30% of sugar beet production in Europe, has been estimated under current limitation of water resources [6]. Drought induces diverse physiological and biochemical changes in crop plants. The first consequence of drought is stomatal closure which can reduce water potential resulting in an inhibition of carbon dioxide uptake, photosynthesis, and subsequent reduction in carbohydrate production and its allocation to the root organ [7]. Another early consequence of low water availability under drought is a decrease in total nutrient uptake and translocation to shoots [8]. This results mainly from a reduced transpirational flow and hence a decreased mass flow to the root surface of soil water containing soluble nutrients like potassium (K), nitrogen (N), and Ca^2+^ [9]. Therefore, it is crucial to maintain the proper uptake of mineral nutrition and functioning of photosynthetic machinery in order to prevent the losses of sugar yield under such a harsh condition.

In general, plants evolve various physiological, morphological, and biochemical responses to cope with drought stress [10]. Among the physiological responses, the protective effect of plant growth regulators and osmoprotectants like free amino acids, sugars, and polyamines, has been well documented [11]. Moreover, the role of mineral nutrition in alleviation of drought stress has widely been shown in crop plants. As an example, foliar application of K together with N and phosphorus (P) enhanced grain yield in wheat under drought stress [12]. Sugar beet has the highest nutrient demands. Besides the main nutrients such as N, P, K, magnesium (Mg), and sulfur (S), sugar beet also needs trace minerals such as boron and manganese. Among these minerals, K and Mg play vital roles in sugar beet growth and development due to their involvement in the photosynthetic activity, sugar synthesis, and its efficient transport into the beetroot as storage organ [13,14]. In addition, as an essential plant nutrient, Ca^2+^ plays a vital role in plant growth and development, like structural roles in the cell wall and membranes, counter-cation for inorganic and organic anions in the vacuole, and as an intracellular messenger in the cytosol [15]. Increasing evidences have also indicated the importance of Ca^2+^ in the regulation of photosynthesis [15,16,17,18,19,20]. In these studies, the function of the involved proteins in photosynthesis machinery as well as their Ca^2+^ dependency have been investigated. It is worthy to note that the role of Ca^2+^ in alleviating drought stress has also been studied in different plants like *Arabidopsis thaliana* [21] and maize [22]. So far, this relation has rarely been addressed in sugar beet, leaving open whether and to what extent the Ca^2+^ nutritional status protects carbohydrate metabolism and loss of sugar in beetroot from drought stress and how it might modulate the regulation of the primary metabolites and impact on the expression of the genes involved in sugar transport mainly SWEET-type and SUT/SUC transporters.

Therefore, to address the role of Ca^2+^ in drought stress in sugar beet, a sugar beet genotype was selected and submitted to drought stress in order to evaluate the effectiveness and relation of a Ca-product and stress tolerance in sugar beet. We hypothesized that drought stress would negatively impact plant growth and metabolism and inhibit the loading of sucrose to the beetroots, thus lowering sugar yield. The foliar application of Ca^2+^ to drought-stressed sugar beet, enhances the chlorophyll level and plant biomass, increases the synthesis of sucrose in leaves and its loading to storage root, thus mitigating drought stress responses. Therefore, investigating plant growth parameters as well as plant mineral status and changes in primary metabolites pointed to a vital role of Ca^2+^ under drought stress.

## 2. Results

### 2.1. Foliar Application of Ca^2+^ Promoted Sugar Beet Growth, Chlorophyll Concentration, Plant Height, and Leaf Coverage Area under Drought Stress

**Figure 1:**
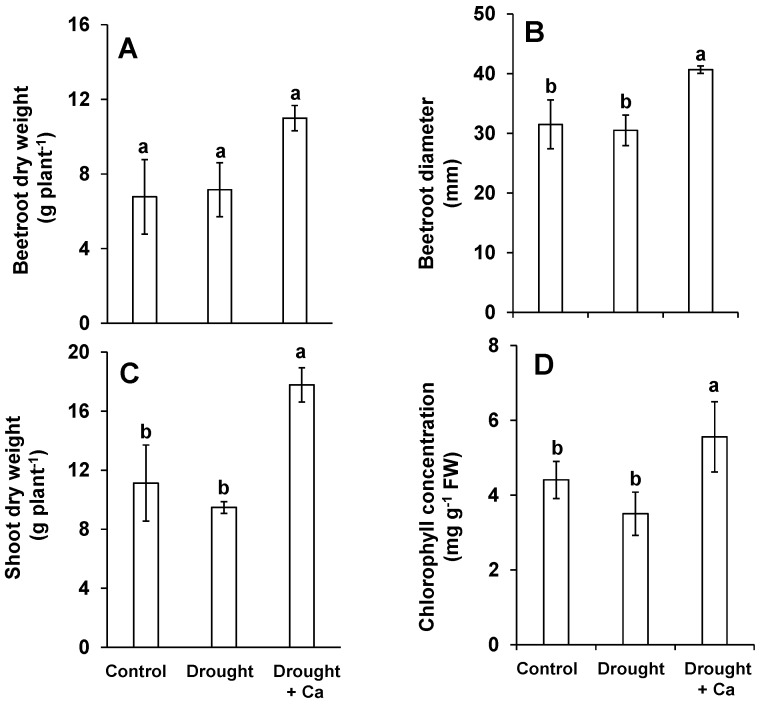
Influence of foliar application of Ca^2+^ on sugar beet root and shoot biomass and chlorophyll concentration under drought stress. (**A**) Beetroot dry weight; (**B**) beetroot diameter; (**C**) shoot dry weight; (**D**) chlorophyll concentration. Plants were grown in pots for a duration of 8 weeks. Five-week-old sugar beet plants were kept at 90% field capacity as control or exposed to drought stress (30% field capacity) for a duration of 3 weeks. Ca^2+^ was applied at BBCH14 and BBCH18 in concentration of 5 L ha^−1^ corresponding to 540g Ca ha^−1^. Leaves and beetroot were harvested at 60 days after sowing for biomass and chlorophyll analysis. Bars indicate means ± SD. Different letters denote significant differences according to ANOVA followed by SNK test (*p* < 0.05; *n* = 6).

The impact of water stress was evaluated through a set of parameters related to growth, measured on beetroot and leaves at 35 days after first Ca^2+^ application (DAA). Drought stress did not provoke a particular change in beetroot dry weight (DW) as well as beetroot diameter compared to the control plants (Figure 1A,B). Interestingly, the foliar application of Ca^2+^ increased beetroot dry weight (54%) under drought stress compared to control and drought stress alone (Figure 1A). This increase was also statistically significant for beetroot diameter (Figure 1B). In shoot, except a slight decrease in dry weight, the imposition of drought alone did not have any significant effect on shoot dry weight compared with that in control (Figure 1C). This slight decrease in shoot dry weight between control and drought stress encouraged us to verify that drought stress was sufficiently applied in our trial. Therefore, the expression levels of choline monooxygenase *(BvCMO)* and dehydration responsive element-binding 2A *(BvDREB2A)* as drought stress markers in sugar beet were examined. Expression of both genes were induced under drought stress alone compared to control (Supplementary Figure S1) displaying that drought stress was sufficiently induced. Like in beetroot, the foliar application of Ca^2+^ significantly increased the shoot dry weight in comparison with control and drought alone (Figure 1C). Next, the level of chlorophyll was determined as a physiological marker for drought stress. The chlorophyll concentration was slightly decreased in drought-stressed plants in comparison to control (Figure 1D). Nevertheless, the foliar application of Ca^2+^ significantly augmented the level of chlorophyll compared to control and drought stress alone (Figure 1D).

**Figure 2:**
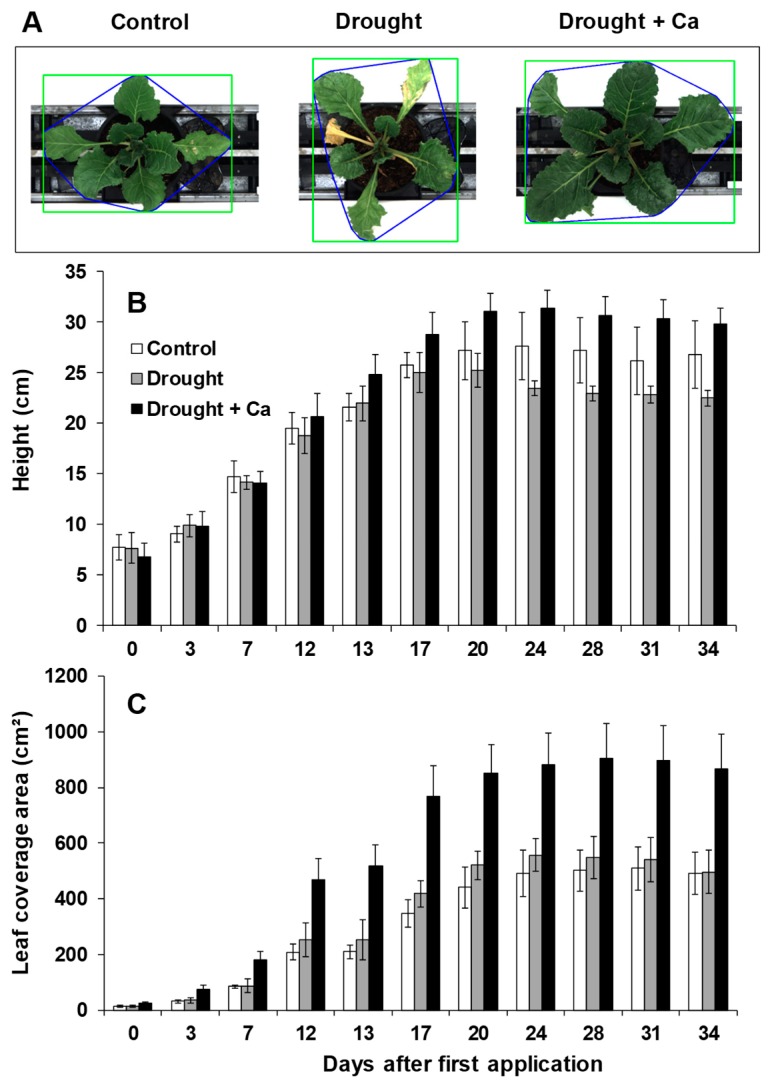
Influence of foliar application of Ca^2+^ on sugar beet leaf coverage area shape and height under drought stress. (**A**) Representative image-based detection of leaf coverage area; (**B**) plant height estimation based on lateral imaging; and (**C**) leaf coverage area. Plants were grown in pots for a duration of 8 weeks. Five-week-old sugar beet plants were kept at 90% field capacity as control or exposed to drought stress (30% field capacity) for a duration of 3 weeks. Ca^2+^ was applied at BBCH14 and BBCH18 in concentration of 5 L ha^−1^ corresponding to 540 g Ca ha^−1^. Plants were imaged every four days from first Ca^2+^ application. Bars indicate means ± SD.

During plant growth and every 4 days after application of Ca^2+^, both plant height and leaf coverage area were monitored in Roullier’s high-throughput plant phenotyping platform (Figure 2A). Plant height did not differ between control and drought stress alone up to 20 DAA of Ca^2+^ (Figure 2B). From day 24, the plant height tended to decline under drought stress alone which reached to the maximum reduction at day 34 after Ca^2+^ application (Figure 2B). The positive effect of Ca^2+^ on plant height was already visible at 12 DAA of Ca^2+^, where this trait was positively increased in drought-stressed plants up to the end of trial (Figure 2B). Moreover, the leaf coverage area was increased under drought stress from the beginning of foliar application of Ca^2+^ (3 DAA) compared to control and drought alone and this increase was accelerated at 24 DAA till end of the experiment (Figure 2C). Altogether, these results indicate that foliar application of Ca^2+^ has a positive effect on biomass of sugar beet subjected to drought stress.

### 2.2. Foliar Application of Ca^2+^ Increased the Concentration of Magnesium (Mg) and Silicon (Si) in Sugar Beet Leaves under Drought Stress

**Figure 3:**
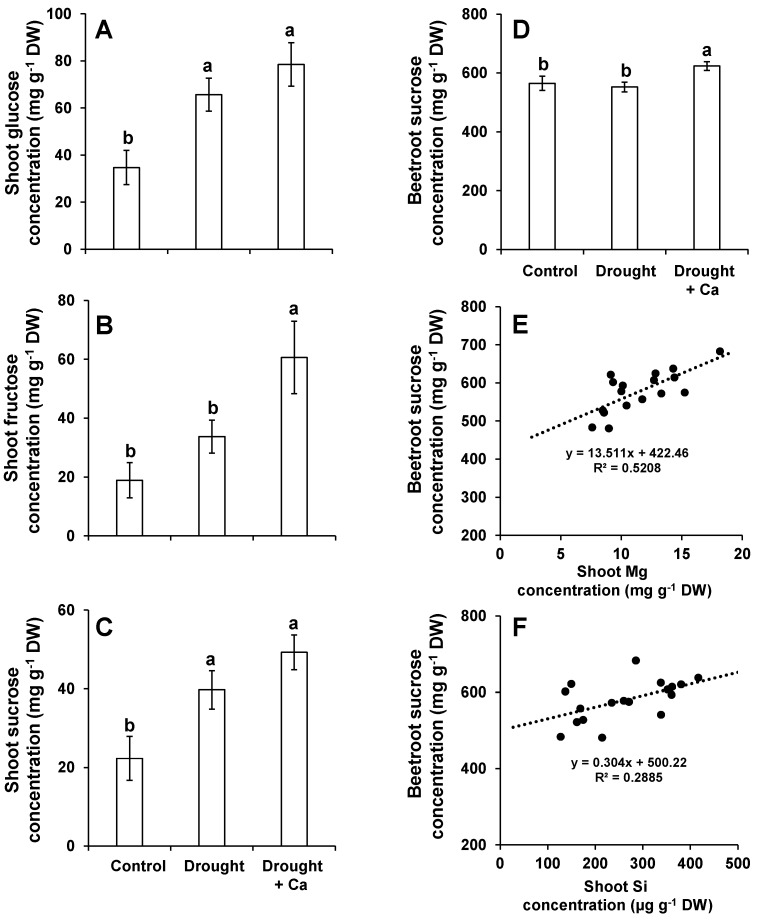
Influence of foliar application of Ca^2+^ on soluble sugar concentration in sugar beet exposed to drought stress. (**A**) Shoot glucose concentration; (**B**) shoot fructose concentration; (**C**) shoot sucrose concentration; (**D**) beetroot sucrose concentration; (**E**) correlation between beetroot sucrose content and shoot Mg content; and (**F**) correlation between beetroot sucrose content and shoot Si content. Plants were grown in pots for a duration of 8 weeks. Five-week-old sugar beet plants were kept at 90% field capacity as control or exposed to drought stress (30% field capacity) for a duration of 3 weeks. Ca^2+^ was applied at BBCH14 and BBCH18 in concentration of 5 L ha^−1^ corresponding to 540 g Ca ha^−1^. Leaves and beetroot were harvested at 60 days after sowing for soluble sugars analysis. Bars indicate means ± SEM. Different letters denote significant differences according to ANOVA followed by SNK test (*p* < 0.05; *n* = 6).

To evaluate the nutritional status of sugar beet plants in response to foliar application of Ca^2+^, elemental profiling was performed for both beetroot and leaf samples. We did not observe any particular changes for both macro and micro-elements in beetroot of both control and drought-stressed plants (Table 1). In general, the level of macro-elements did not fall below the optimum needs of sugar beet. We did not observe any particular changes in the level of N in both beetroot and leaf organs under drought (Table 1). The remarkable changes in the nutritional analysis were found in drought-stressed plants when Ca^2+^ was sprayed. Among macro-elements, foliar application of Ca^2+^ significantly decreased the concentrations of both P and K in beetroots compared to the control and drought-stressed plants which were not sprayed with Ca^2+^ (Table 1). This trend also held true for micro-elements iron, manganese, zinc, and copper. In leaves, concentrations of both P and K were decreased in drought-stressed plants with foliar application of Ca^2+^ compared to both control and drought alone (Table 1). We did not observe any consistent changes in the levels of beetroot Mg and Si with the foliar application of Ca^2+^ (Table 1). However, a significant increase in the levels of Mg and Si was observed in the leaves of drought-stressed plants in comparison to control and drought alone (Figure 3A,B). Like N, the concentration of Na did not change in the current study. However, due to an important role of Na in sugar beet growth, the Na/K ratio was calculated in both beetroot and leaf (Table 1). The changes were not statistically confirmed, however, in leaf and under drought stress, the Na/K ratio increased nearly 40% compared to control and drought stress alone (Table 1).

We also observed a significant reduction in the levels of both zinc and copper under drought stress when Ca^2+^ was supplied (Table 1). The results showed that the foliar application of Ca^2+^ decreased the accumulation of mineral nutrients in the beetroot while it increased the level of macro-elements Mg and Si in leaves.

### 2.3. Foliar Application of Ca^2+^ Increased the Concentration of Sugars in Beetroot and Leaves Exposed to Drought Stress and Induced the Expression of the Genes Involved in Sugar Transport

In the next approach, the concentrations of soluble sugars, particularly sucrose (Suc) as a major economical part of sugar beet, were determined. In general, drought stress alone increased the levels of leaf Glu, Fru, and Suc compared to the control and this increase was not statistically significant for Fru (Figure 3A–C). Interestingly, under drought condition, foliar application of Ca^2+^ increased the level of Glu, Fru, and Suc by 20%, 80%, and 24%, respectively, when compared to drought alone (Figure 3A–C). The beetroot Suc levels did not differ between control and drought-stress plants, while foliar application of Ca^2+^ significantly increased its level by 13% under drought stress (Figure 3D).

Given that Suc concentration increased in beetroot, we analyzed the correlation between beetroot sucrose concentration and both Mg and Si levels in leaves. The positive correlation was observed between Mg concentration in leaves and Suc concentration in beetroot (*R*^2^ = 0.52) (Figure 3E). This correlation was also positive in a lesser extent for Si concentration in leaves and Suc concentration in beetroot (*R*^2^ = 0.28) (Figure 3F).

**Figure 4:**
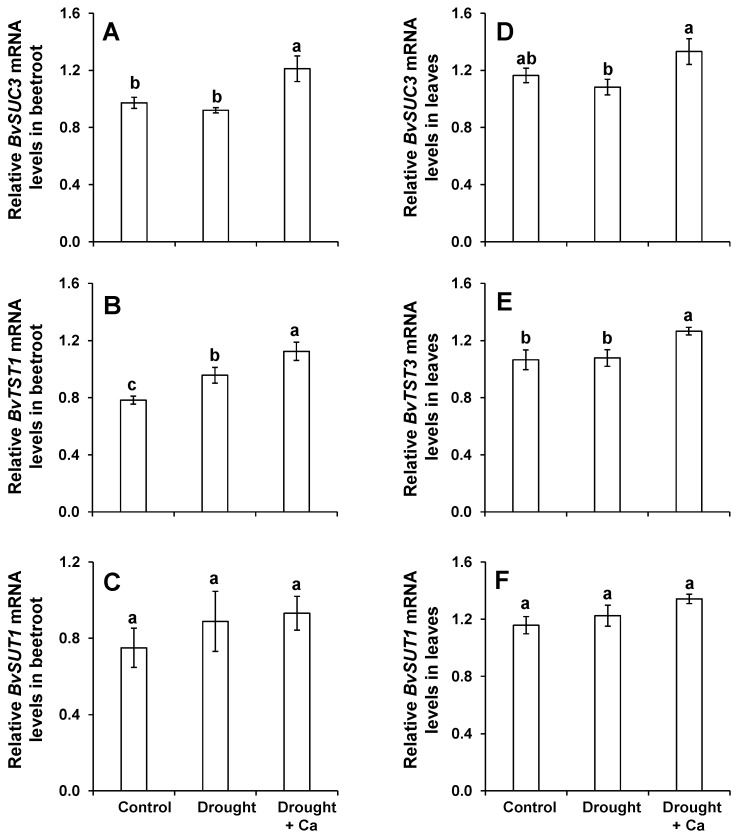
Influence of foliar application of Ca^2+^ on the expression levels of the genes involved in sugar transport in sugar beet plants exposed to drought stress. (**A**) Relative *BvSUC3* mRNA levels in beetroot; (**B**) relative *BvTST1* mRNA levels in beetroot; (**C**) relative *BvSUT1* mRNA levels in beetroot; (**D**) relative *BvSUC3* mRNA levels in leaves; (**E**) relative *BvTST3* mRNA levels in leaves; and (**F**) relative *BvSUT1* mRNA levels in leaves. Plants were grown in pots for a duration of 8 weeks. Five-week-old sugar beet plants were kept at 90% field capacity as control or exposed to drought stress (30% field capacity) for a duration of 3 weeks. Ca^2+^ was applied at BBCH14 and BBCH18 in concentration of 5 L ha^−1^ corresponding to 540 g Ca ha^−1^. Leaves and beetroot were harvested at 60 days after sowing for gene expression analysis. Bars indicate means ± SEM. Different letters denote significant differences according to ANOVA followed by SNK test (*p* < 0.05; *n* = 6).

The impact of foliar spray of Ca^2+^ on drought-stressed sugar beet was also evaluated at the molecular level by monitoring the expression of the genes related to phloem and cellular export/loading of Suc, SWEET-type, and SUT/SUC transporters. In beetroots, the expression level of *BvSUC3* was approximately the same in control and drought stress alone (Figure 4A), whereas the expression level of this gene was significantly up-regulated by foliar application of Ca^2+^. In addition, the expression level of *BvTST1* was significantly induced under drought stress irrespective of the foliar application of Ca^2+^ and this induction was more pronounced in drought-stressed plants supplied with Ca^2+^ (Figure 4B). We did not observe any particular changes in the expression level of *BvSUT1* in beetroot (Figure 4C). In leaves, transcript levels of *BvSUC3* and *BvTST3* did not differ between control and drought alone, while Ca^2+^ treatment significantly up-regulated the expression of both genes (Figure 4D,E). Moreover, we did not observe any significant differences for the expression pattern of *BvSUT1* under drought stress irrespective of foliar Ca^2+^ application (Figure 4F). Nevertheless, *BvSUT1* expression was slightly, but not significantly, up-regulated under drought in Ca-treated plants compared to control plants (Figure 4F).

### 2.4. Foliar Application of Ca^2+^ Modulated Plant Primary Metabolites under Drought Stress

**Figure 5:**
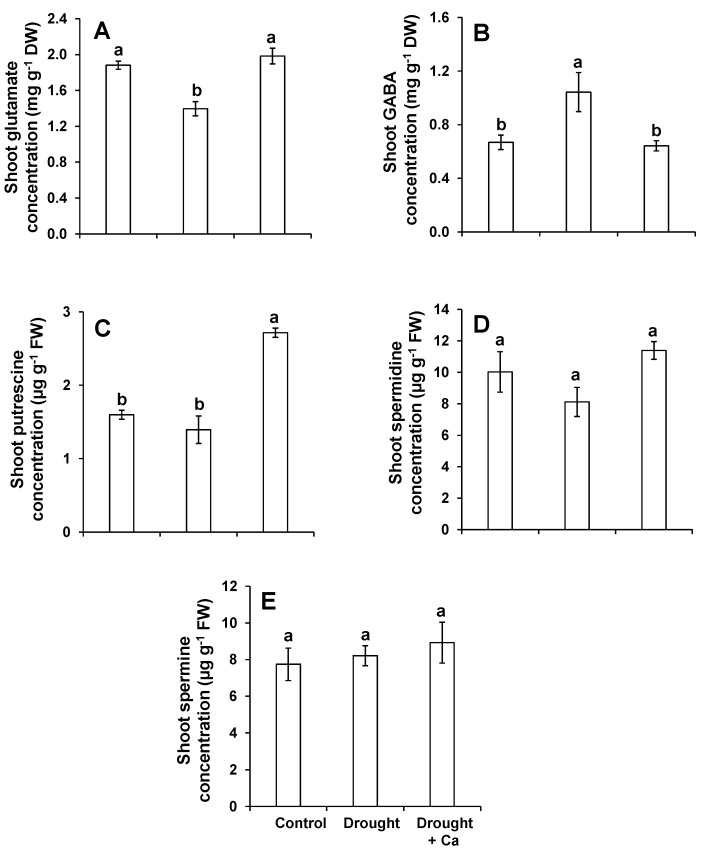
Influence of foliar application of Ca^2+^ on shoot glutamate, GABA, and polyamine concentrations in sugar beet plants exposed to drought stress. (**A**) Shoot glutamate concentration; (**B**) shoot GABA concentration; (**C**) shoot putrescine concentration; (**D**) shoot spermidine concentration; and (**E**) shoot spermine concentration. Plants were grown in pots for a duration of 8 weeks. Five-week-old sugar beet plants were kept at 90% field capacity as control or exposed to drought stress (30% field capacity) for a duration of 3 weeks. Ca^2+^ was applied at BBCH14 and BBCH18 in concentration of 5 L ha^−1^ corresponding to 540 g Ca ha^−1^. Leaves and beetroot were harvested at 60 days after sowing for metabolite analysis. Bars indicate means ± SEM. Different letters denote significant differences according to ANOVA followed by SNK test (*p* < 0.05; *n* = 6).

Because of the important role of metabolites in drought stress tolerance, the effect of Ca^2+^ was further investigated on primary metabolites, mainly those compounds which are involved in stress responses. In beetroots, and compared to control plants, the concentration of glutamate (Glu) was decreased under drought irrespective of foliar application of Ca^2+^ (Figure 5A). While, a significant increase in the levels of glutamine (Gln), lysine (Lys), and leucine (Leu) was observed in drought-stressed plants which were sprayed with Ca^2+^ when compared to control and drought alone (Supplementary Table S1). Also, the levels of isoleucine (Ile), tryptophan (Trp) (Supplementary Table S1), and gamma-aminobutyric acid (GABA) (Figure 5B) were increased under drought in spite of the foliar application of Ca^2+^ and this increase was statistically significant compared to the control plants. In leaves and unlike the beetroots, a positive significant impact of Ca^2+^ on the levels of Glu was observed in comparison to drought alone (Figure 5A and Supplementary Table S1). Moreover, the levels of histidine (His), Trp, and GABA was significantly increased under drought alone, but decreased to the same levels of the control in drought-stressed plants treated with Ca^2+^ (Figure 5B and Supplementary Table S1).

Among the beetroot organic acids, the concentration of citrate was significantly decreased under drought stress in the presence of Ca^2+^ and in comparison to drought alone (Supplementary Table S1). In leaves, the level of isocitrate was significantly increased with foliar application of Ca^2+^ compared to the other treatments (Supplementary Table S1). In addition, the level of malate was increased under drought stress irrespective of the foliar application of Ca^2+^ in comparison to the control plants (Supplementary Table S1).

Monitoring the level of polyamines, metabolites, which are involved in stress responses in plants [23], showed a significant increase in the concentration of putrescine in leaves with foliar application of Ca^2+^ compared to control and drought stress alone (Figure 5C). We did not observe any significant changes in the levels of leaf spermidine and spermine among the tested conditions (Figure 5D,E).

### 2.5. Foliar Application of Ca^2+^ Regulated the Glutathione Pool under Drought Stress

**Figure 6:**
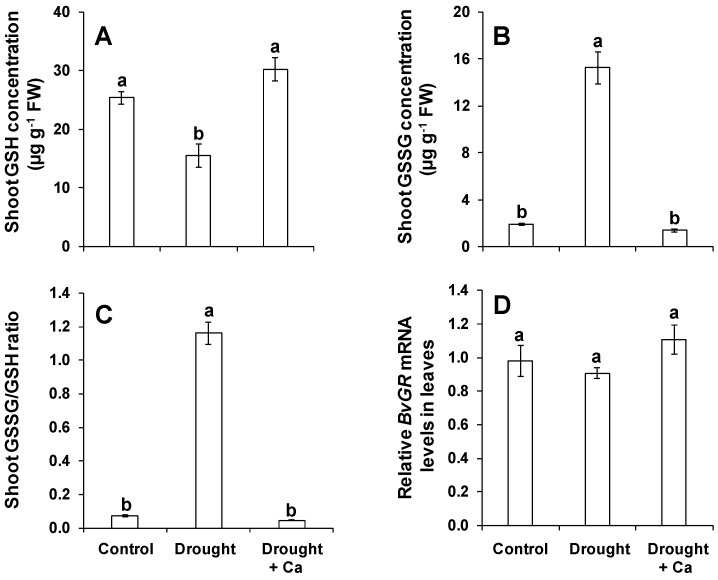
Influence of foliar application of Ca^2+^ on shoot glutathione pool and on the expression of glutathione reductase in sugar beet exposed to drought stress. (**A**) Shoot GSH concentration; (**B**) shoot GSSG concentration; (**C**) shoot GSSH/GSH ratio; and (**D**) relative *BvGR* mRNA levels in leaves. Plants were grown in pots for a duration of 8 weeks. Five-week-old sugar beet plants were kept at 90% field capacity as control or exposed to drought stress (30% field capacity) for a duration of 3 weeks. Ca^2+^ was applied at BBCH14 and BBCH18 in concentration of 5 L ha^−1^ corresponding to 540 g Ca ha^−1^. Leaves and beetroot were harvested at 60 days after sowing for glutathione analysis. Bars indicate means ± SEM. Different letters denote significant differences according to ANOVA followed by SNK test (*p* < 0.05; *n* = 6).

Balancing the redox state via glutathione pool is very critical under drought induced-oxidative stress [11]. Therefore, we measured both reduced (GSH) and oxidized (GSSG) glutathione, and calculated the GSSG/GSH ratio in leaves of control, drought stress alone and drought stress plants which did receive Ca. The GSH concentration was decreased under drought when compared to the control condition (Figure 6A). Interestingly, foliar application of Ca^2+^ significantly enhanced the concentrations of GSH in leaves under drought stress to a similar level of that in control (Figure 6A). As expected, the concentration of GSSG and its ratio to GSH (GSSG/GSH) were increased under drought alone compared to the control (Figure 6B,C). However, Ca^2+^ treatment strongly reduced their level to the same level of control plants (Figure 6B,C). We further examined the expression level of glutathione reductase (*BvGR*) as a key gene for recycling the GSSG to GSH for balancing the redox potential under stress conditions [11]. The expression of this gene was slightly, but not significantly, induced under drought stress by foliar spray of Ca^2+^ as compared to drought stress alone (Figure 6D). These results indicate that foliar application of Ca^2+^ mitigates drought stress-induced oxidative stress in sugar beet.

## 3. Discussion

Strong evidences have demonstrated that plant mineral nutrition plays a vital role in mitigating drought stress in crop plants [8,24]. However, despite the important signaling role of Ca^2+^ in plant responses to different abiotic stresses [25], research for the beneficial role of Ca^2+^ in drought stress tolerance in sugar beet is not carefully considered. In our study, monitoring the responses of sugar beet to drought stress and foliar application of Ca^2+^ showed that the higher Suc in beetroot is associated with higher Mg and Si in leaves which positively augments the chlorophyll level and shoot biomass and increases the synthesis of soluble sugars. We clearly show that the application of Ca^2+^ to sugar beet plant could be a useful strategy to increase drought tolerance and to decrease beetroot sugar losses.

### 3.1. Foliar Application of Ca^2+^ Increases Mg and Si Levels in Leaves and Promotes Plant Growth, Height, Leaf Coverage Area Parameters under Drought Stress

The main consequence of drought stress is the restriction of plant growth and development. Increasing evidences have shown a growth reduction under drought stress in maize [26], and in other plants like *A. thaliana* [27], wheat [28], and rice [29]. In addition, the sensitivity of sugar beet to limited water supply has been previously reported [5,6,7]. For instance, Hoffmann et al., (2010) showed a reduction in both shoot and root dry matter in sugar beet subjected to drought stress [4]. They showed that the reduction in dry matter was more pronounced in the root organ. In the present work, both beetroot and shoot biomass as well as beetroot diameter responded positively to Ca^2+^ application under drought stress. Interestingly, the positive effect of Ca^2+^ was also held true for plant height and leaf coverage area. These results are in line with previous work on sugar beet, where the foliar application of both Si and Ca^2+^ increased the root and shoot biomass by 13% and 21%, respectively [30]. The higher biomass in this study can be explained by the role of Ca^2+^ in stabilizing the structure of the organelles in photosynthetic machinery under drought condition [31].

Water scarcity also leads to a decrease in total nutrient uptake and translocation to the shoots [8]. Under such a condition, the plant’s normal metabolism is restricted, which ultimately reduces crop yields [32]. Plant leaves have capacity to absorb water and nutrients, however, foliar spray of nutrients can be a good strategy to maintain crop yields and quality under conditions of limited soil nutrient as well as lack of available water [9]. In a study on *Citrus macrophylla* L. seedling, foliar application of potassium nitrate improved the tolerance to drought stress [33]. Also, exogenous Ca^2+^ was shown to alleviate the degree of photosynthesis pigment degradation and ensured normal photosynthesis in tobacco plant subjected to drought stress [34]. In our elemental profiling, Ca^2+^ application modulated distinct changes on the level of macro-elements: while the levels of N, Ca, S, and Na did not differ in beetroot and leaf, concentrations of both K and P decreased in beetroot and leaf by application of Ca^2+^. The decrease in the level of beetroot K and P may be due to a decrease in transpirational flow and hence a decreased mass flow and diffusion-driven nutrient transport under drought stress even if it was not evident in drought stress treatment alone [9,35]. In plants, a high proportion of the total Ca^2+^ is mainly located in the cell walls. Compared to monocotyledons, sugar beet has a large cation-exchange capacity as dicotyledon crops. Upon higher Ca^2+^ supply, excess Ca^2+^ is generally accumulated in the vacuole. This is similar in the case of K where vacuolar concentrations of K may reach up to 500 mM [36]. The K concentration in the vacuoles can be replaced by other cations (Na^+^, Mg^2+^, Ca^2+^) explaining why application of Ca^2+^ reduced the level of K in drought-stressed leaves in the present work [9]. Additionally, the lower concentrations of K and P in the leaf can be the result of lower uptake and translocation of these nutrients under drought condition which may cause uptake of other cations like Mg in non-specific cation absorption manner. Apparently, allocation of Ca^2+^ and P to separate cell types avoids the negative effect of precipitation of Ca-P, which would otherwise decline the availability of both nutrients [37]. Nevertheless, one might hypothesize that the observed decrease in the levels of leaf K and P after Ca^2+^ application serves potentially as a negative impact on the plant’s growth. Conversely, looking to the average concentrations of K (>1%) and P (0.3–0.6%) in leaf, an adequate range of both elements for optimal growth and development of plant was observed.

Na is another indispensable element which has stimulation effect on leaf growth in sugar beet. The role of Na in improving water relation under drought stress has also been well investigated [9]. The positive function of Na in response to drought stress was linked to its higher absorption under drought condition [38]. It has been shown that drought stress in *Triplex canescens* and *Sesuvium portulacastrum* was due to absorption of Na which was used directly for osmotic adjustment [38,39]. The involvement of Na in regulation of osmotic adjustment has been also shown in sugar beet plants imposed with osmotic stress [40]. In sugar beet, Na can be replaced by K to a large degree. The positive effect of Na on yield of sugar beet was shown in suboptimal concentration of K [41]. Hence, considering the low concentration of K under drought stress which was induced by Ca^2+^ supply, we assume that Na might play a role even if its levels did not change. This was confirmed by higher Na/K ratio in shoot (~40%) indicating the possible role of Na in osmotic adjustment under drought stress and explaining to some extent the low concentration of K in leaf under drought by Ca^2+^ application.

As an indispensable divalent cation, Mg plays many vital roles in plants [42]. Mg is involved in energy metabolism and acts as an enzyme cofactor, carbohydrate partitioning, and enzyme activation [2,42]. It is also involved in the central atom of the chlorophyll molecule [9]. Under Mg deficiency, the necrosis spots appear in the young leaves due to the transport of Mg from old leaves, and thus breakdown of both plastid pigments and chlorophyll are increased [13,43]. In the present study, application of Ca^2+^ showed an increase in the level of Mg in the leaves which positively impacted on the levels of chlorophyll. This finding is in good agreement with a study in oregano (*Origanum vulgare ssp. hirtum*), where foliar application of Ca^2+^ increased the level of chlorophyll [44]. Ca-increased Mg concentration in leaf can be due to low concentration of leaf K as these two cations can be easily replaced [9]. This hypothesis was supported by a lower Mg/K ratio in leaf (data not shown). Therefore, the higher leaf Mg induced by high Ca^2+^ and low K levels in one hand increased the chlorophyll level which maintained the photosynthetic machinery active. The proper functioning of photosynthesis led to substantial synthesis of soluble sugars in leaf and its translocation to beetroots, thus translated to higher biomass.

Si is another reported beneficial nutrient which has been shown to increase drought stress tolerance and mitigate mineral nutrient deficiency in crop plants [45]. For example, Si has been shown to increase root and shoot biomass under drought condition in xerophyte *Zygophyllum xanthoxylum* [46], tomato [47], and barley plants [24,48]. Moreover, foliar application of Si to *Sorghum bicolor* (L.) plant exposed to drought was shown to maintain a higher stomatal conductance and enhanced drought tolerance [49]. Numerous studies have also shown that Si addition to stressed plants significantly increased chlorophyll concentration and delayed leaf senescence [46,50,51,52]. In line with the above-mentioned studies, our study showed higher concentration of chlorophyll under drought after Ca^2+^ supply which could be due to an increase in the levels of leaf Si. It is worth to note that Ca^2+^ application might have an indirect effect on leaf Si status and cross talk between these two elements and needs to be further experimentally investigated.

Altogether, our finding reveals that foliar application of Ca^2+^ had a positive effect on sugar beet plants under drought condition by mediating distinct changes in the levels of nutrients like Mg and Si in leaves, which caused an increase in chlorophyll and sugar levels and eventually enhanced plant growth and biomass.

### 3.2. Foliar Application of Ca^2+^ Increases the Level of Soluble Sugars in Leaves and Beetroots and Induces the Genes Involved in Sucrose Transport

The delivery of assimilates which results from leaf growth and photosynthesis is crucial for the accumulation of sucrose in storage root of sugar beet. In this regard, the improvement of plant growth by application of Ca^2+^ has further encouraged us to evaluate the changes in concentration of soluble sugars in the leaves and in the beetroot. A considerable reduction in the sucrose accumulation of beetroot in drought-stressed sugar beet has been already reported [53,54]. As an example, the work of Mäck and Hoffmann (2006) on sugar beet exposed to drought stress showed a reduction in sucrose concentration in beetroot due to the contribution of ions and other compatible solutes rather than a limitation in physical structure such as cambium rings [55]. To cope with drought stress, plants accumulate different compatible solutes, like amino acids or sugars to allow efficient osmotic adjustments [56,57]. The higher accumulation of Suc in the leaves of sugar beet as a result of drought stress can change the phloem loading process [55], which then induces leaf senescence, restricts water loss via transpiration [58], and downregulates photosynthesis [59]. In the present work, Ca^2+^ remarkably increased the concentrations of leaf soluble sugars Fru and particularly Suc under drought stress. Here, we also observed a significant increase (13%) in beetroot Suc concentration under drought by foliar application of Ca^2+^. The higher levels of carbohydrate in leaves could be beneficial since it also serves an osmoticum under drought stress. Moreover, increased levels of sugars in the leaves and the loading of Suc to the beetroot can also be explained by the higher concentration of Mg and Si in the leaves. Indeed, previous studies have indicated that the shortage of Mg may restrict phloem loading of source in leaves resulting in growth constraint, particularly in roots [2,60,61,62]. However, in our work, the foliar application of Ca^2+^ resulted not only in higher concentrations of leaf Mg, but also simultaneously enhanced the beetroot Suc concentrations. We also found that among the other solutes (K, Na, amino acids), Suc was the major compound which accumulated in beetroot under drought condition when foliar application of Ca^2+^ was applied. Another explanation for higher Suc in beetroot could be due to the higher concentration of leaf Si. We have shown in our previous works in barley plants subjected to concomitant drought and mineral deficiencies (low K and low sulfur) that Si supply to the roots increased the sugar levels in shoots and allowed the loading of Suc into the roots [24,48]. In our earlier work, we have also shown that Si supplement, increased the levels of Si in maize plants grown under low Mg which turned to higher levels of soluble sugars in both leaves and roots [52]. Altogether, our findings indicate that foliar application of Ca^2+^ to drought-stressed sugar beet, increased the levels of Mg and Si in leaves underlying the capacity of the plants to produce more sugars in leaves as osmoticum which simultaneously loads the Suc into the beetroots.

The majority of Suc in taproot of sugar beet is stored in the vacuole [3] by carbohydrate partitioning where sugars are distributed from source leaves to the sink organs like roots [63]. This sucrose is like a reserve for plant growth and development and for synthesis of metabolite and energy particularly when plants are faced with biotic and abiotic stresses. Delivery of Suc within the plant cell is carried out with different sugar transporters mainly SWEET-type and SUT/SUC transporters [63]. In sugar beet, Jung et al. (2015) showed that *BvTST2.1* correlated with the peak of higher Suc accumulation in the taproot [3]. This correlation has also been shown in other crop plants like sugarcane [64] and sweet sorghum [65]. Recently, in a study of *A. thaliana* plants grown in soil-based system, Durand et al., 2016 showed that drought stress induced the expression level of Suc phloem loading genes *AtSWEET11*, *AtSWEET12,* and *AtSUC2* in the leaves [66]. These authors also showed an induction in the expression level of *AtSUC2* and *AtSWEET11* to *AtSWEET15* in the roots. In line with the above-mentioned works, we showed that the expression levels of *BvSUC3* and *BvTST1* induced in beetroot under drought stress with foliar application of Ca^2+^. The positive impact of Ca^2+^ on the expression pattern of *BvSUC3* and *BvTST3* was also detected in leaves. This indicates the putative role of Ca^2+^ in transcriptionally regulating the sugar transporters by allocating more Suc to the beetroot under drought condition.

### 3.3. Foliar Application of Ca^2+^ Enhances Drought Tolerance in Sugar Beet by Regulating Glutathione Pools and Increasing Polyamine Putrescine

Drought stress is considered to imply oxidative stress by disturbing the equilibrium between production of reactive oxygen species (ROS) and their detoxification [67]. Previous research has clearly demonstrated that plants exposed to drought stress triggered reactive oxygen species (ROS) production which ultimately impacts plant growth and development as a result of cell death [67,68]. However, plants evolve enzymatic and non-enzymatic defense mechanisms to cope with oxidative stress [69]. In addition, the regulation of ascorbate-glutathione cycle was also found to increase oxidative stress tolerance in different crop plants [70,71]. Under suboptimal water supply, the elevated levels of ROS result in the increase of oxidized glutathione (GSSG) in favor of reduced glutathione (GSH), thus inducing a higher GSSG/GSH ratio and results to severe oxidative stress [11,72]. The reduction in glutathione pools under drought stress has been shown in different crops like wheat [73], maize [70], and rice [74]. In line with the above-mentioned studies, the level of GSH significantly reduced under drought stress alone with a tandem increase in the concentrations of GSSG resulting in a higher GSSG/GSSG ratio. However, foliar application of Ca^2+^ significantly increased the level of GSH to the similar level of the control plants and sharply reduced the GSSG/GSH ratio under drought. This observation was interesting, since a low GSSG/GSH ratio is a critical factor under drought stress. This was further supported by the slight induction in *BvGR* expression as a key factor for recycling the GSSG to GSH for balancing the redox state under stress conditions [11]. Our finding is also supported by our previous investigation in barley where root coped better with oxidative stress by implying a lower GSSG/GSH ratio and higher production of GSH [24]. This indicates that foliar application of Ca^2+^ positively balanced the glutathione pools and cellular redox homeostasis under drought stress which implicated lower oxidative stress.

The Ca-induced drought tolerance in sugar beet was further evaluated with regards to metabolite changes by focusing mainly on the polyamine pathway as well as amino acids. The role of polyamines in plant stress tolerance has been well reported in several studies [23,75]. In the present study, and among the polyamines, foliar application of Ca^2+^ significantly increased the levels of putrescine under drought stress. The higher concentration of putrescine can be associated with increased levels of Si when Ca^2+^ was sprayed to the drought-stressed sugar beet plants. We have shown in our previous works that Si modulates polyamines pathways in response to combined stresses [24,48] and Mg deficiency [52]. Moreover, we have reported that the application of Si to a drought-sensitive tomato line increased the levels of polyamines putrescine and spermine, leading to drought stress tolerance [47]. The cross talk between Si and polyamine pathways has also been shown in the Sorghum plant exposed to drought stress [76] where the levels of free putrescine, spermidine, and spermidine increased at the expense of ethylene.

It is also worth noting that in the leaves, the concentration of GABA enhanced significantly under drought stress. This increase was expected as the higher production of GABA is a common physiological response to drought stress [77]. However, foliar application of Ca^2+^ declined the GABA level under drought stress. These results indicated that sugar beet plants which were supplied with Ca^2+^ did not face severe stress and withstood water stress better than drought stress alone. Meanwhile, the concentration of glutamate (Glu) significantly increased under drought by foliar application of Ca^2+^. Both GABA and proline are synthesized from Glu which is the main pathway for their production under drought condition [77]. Glu is also involved in the production of free polyamines [78]. Notably, we have shown in our previous work that Si improved osmotic stress tolerance in drought-sensitive tomato line by increasing the production of Glu which was associated with higher synthesis of amino acid GABA and polyamine Put [47]. Hence, in the present work, the higher level of Glu by foliar application of Ca^2+^ could be due to the subsequent increase in the leaf Si. Moreover, it seems, at least in our experimental condition, Glu influenced polyamine synthesis rather than production of stress amino acid GABA. This could be further supported as we did not observe any consistent changes in the level of proline. These results, on one hand, indicate that the Ca-treated plants most probably suffered less from drought and did not require an increase in the level of stress amino acids like GABA. On the other hand, Ca^2+^ allowed plants to cope better with drought stress since it positively modulated the polyamine pathway and increased the level of Put as a result of higher Glu synthesis.

## 4. Materials and Methods

### 4.1. Seed Germination, Plant Material, and Stress Treatment

**Figure 7:**
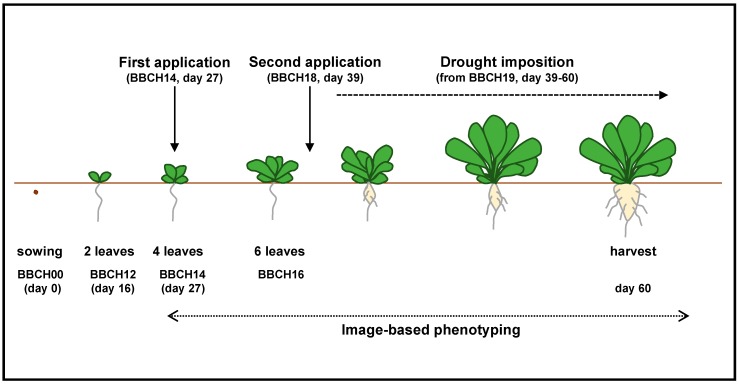
Schematic representation of experimental design. After seed germination, the seedlings were grown for 8 weeks in the Roullier high-throughput plant phenotyping platform. Foliar application of Ca^2+^ was supplied twice at BBCH14 and BBCH18, each time with a dose of 5 L ha^−1^ corresponding to 540 g Ca ha^−1^. Drought stress (30% field capacity) was imposed 2 days after the second application of Ca^2+^ for a duration of 3 weeks, while control plants were kept in 90% of field capacity. From BBCH14 till end of the experiment, image-based phenotyping was applied every 4 days. All plants were harvested at 60 days after sowing for different physiological, biochemical, and molecular analyses.

Seeds of sugar beet (*B. vulgaris* L.) Marinella genotype was kindly provided by KWS via COPROB, Minerbio, Italy. Seeds were germinated on Podground H90 substrate (Klasmann, Bremen, Germany) for 16 days (20/18 °C, 16 h day, 80% relative humidity). The experiment was completely randomized and each treatment was represented by 6 replicate pots with one plant per pot. Plants were cultivated in 6.5 L pots (Pöppelmann, Lohne, Germany) using Substrate 5 GreenFibre (Klasmann). According to manufacturer, this substrate contains 180 mg L^−1^ N, 210 mg L^−1^ P_2_O_5_, 360 mg L^−1^ K_2_O, 100 mg L^−1^ Mg, and 150 mg L^−1^ S. Foliar application of Ca^2+^ was performed five weeks after germination at BBCH14 and BBCH18 in a dose of 5 L ha^−1^ corresponding to 540 g Ca ha^−1^ in each application using automated machine customized for spraying fertilizers (InoviaFlow, Dole, France). This product contained 15% CaO (CaCl_2_). Drought stress (30% field capacity) was imposed 2 days after the second application of Ca^2+^ for a duration of 3 weeks. Control plants were watered continuously and were kept at 90% field capacity (Figure 7).

From BBCH14 (Biologische Bundesanstalt Bundessortenamt und Chemische Industrie) [79] till the end of the experiment, plants were monitored in the Roullier high-throughput plant phenotyping platform (16 h day [in greenhouse equipped with high pressure sodium lights] at 22 °C, and 8 h night at 19 °C with 70% relative humidity in the air) where images of each plant were taken by using two imaging units. The first imaging unit was composed of top and side high definition red-green-blue (RGB) cameras and light-emitting diode (LED) light system (5500 K ± 500 K). The second imaging unit was composed of a thermal camera, 2-CCD (charge-coupled device) camera (near-infrared and color sensors) and LED light systems (5500 K ± 500 K and 850 nm). For all three color images of each plant, we used a customized segmentation algorithm (unpublished) to extract the masks of the plants and remove the background. Mathematical morphology algorithms were applied to filter artefacts according to the quality of the segmentation. Then the masks were applied on the near infrared reflectance (NIR) images and on the thermal files. For the thermal data, an image registration algorithm was used to transform the mask to fit these data. Once all the images were segmented, projected areas for top and side views, as well as the width and height for side views, were calculated. All plants were harvested at 60 days after sowing for different physiological, biochemical, and molecular analyses.

### 4.2. Determination of Root and Shoot Biomass

For determination of biomass, the fresh beetroot was harvested and weighed. One part of the samples was immediately frozen in liquid N_2_ and the other part was dried in an oven at 70 °C for dry matter determination. In parallel, all leaves were harvested and treated the same way.

### 4.3. Determination of Chlorophyll

Chlorophyll concentration was measured according to Connan [80]. Briefly, 25 mg of fresh material was incubated with 0.5 mL of acetone and sonicated for 10 min. After centrifugation at 10,000 rpm at 4 °C for 10 min, the supernatant was transferred to a new tube. A second extraction was performed on the pellet with 0.5 mL of acetone following the same protocol. The two supernatants were collected in the same tube and evaporated under nitrogen flow. Pellet was re-suspended in 1 mL of methanol and the absorbance was determined at 632, 652, 665, and 750 nm.

### 4.4. Determination of Mineral Elements

Elemental analysis was performed according to Maillard et al. [81] in the PLATIN’ (Plateau d’Isotopie de Normandie) core facility. Elements were quantified by high-resolution inductively coupled plasma mass spectrometry (HR ICP-MS, Thermo Scientific, Element 2TM) with prior microwave acid sample digestion (Multiwave ECO, Anton Paar, les Ulis, France) (800 µL of concentrated HNO_3_, 200 µL of H_2_O_2,_ and 1 mL of Milli-Q water for 40 mg DW). For the determination by high resolution inductively coupled plasma mass spectrometry (HR ICP-MS) all the samples were spiked with two internal-standard solutions of gallium and rhodium for final concentrations of 10 and 2 µg L^−1^, respectively, diluted to 50 mL with Milli-Q water to obtain solutions containing 2.0% (*v*/*v*) of nitric acid, then filtered at 0.45 µm using a teflon filtration system (Filtermate, Courtage Analyses Services, Mont-Saint-Aignan, France). Quantification of each element was performed using external standard calibration curves.

Analysis of N was performed using an elemental FLASH 2000 CHNS analyzer (Thermo Scientific, Waltham, MA, USA) according to manufacturer’s instructions from 2.5 mg of homogenized and lyophilized plant material.

### 4.5. Determination of Primary Metabolites

Soluble sugar determination was undertaken according to the method described by Kim et al., 2013 [82]. Ten mg lyophilized shoot material was homogenized in liquid nitrogen, dissolved in 0.75 mL of 80% (*v*/*v*) ethanol, and incubated at 80 °C for 30 min. Crude extracts were decanted for 15 min at retention time (RT), centrifuged at 14,000 rpm for 10 min at 4 °C, and concentrated in a Speed Vac concentrator (Thermo Scientific) at 45 °C for 180 min. The pellet was re-suspended in 0.75 mL deionized water and incubated at 80 °C for 30 min. After centrifugation, the second supernatant was added to the first, concentrated and resuspended in 0.5 mL of double distilled water (ddH_2_O). Hexokinase (HK), phosphoglucoisomerase (PGI), and beta-fructosidase were added successively to measure glucose (Glc), fructose (Fru), and sucrose (Suc), as described in Kim et al., 2013.

For amino acid determination, 10 mg lyophilized dry matter was extracted with a solution containing 400 µL of MeOH and 0.250 nmol/µL Norvaline, which was used as the internal standard (Sigma Aldrich, St. Louis, MO, USA). Extract was stirred for 15 min, and was then re-suspended with 200 µL of chloroform (agitation for 5 min) and 400 µL of ddH_2_O. After centrifugation (12,000 rpm, 10 °C, 5 min), the supernatant was recovered, evaporated, and dissolved in 100 µL of ddH_2_O. Derivatization was performed using an Ultra Derivatization Kit AccQ tag, following the manufacturer’s protocol (Waters Corp, Milford, MA, USA). The amino acid profile was determined by using ultra performance liquid chromatography coupled with photodiode array detector (UPLC/PDA) H-Class system with ethylene bridge hybrid (BEH) C18 100 × 2.1 mm column (pore size: 1.7 µm).

Organic acid analysis was performed as described previously by Ali et al., 2018. Metabolite extraction was conducted using 30 mg of frozen ground fresh leaves and roots, which were weighed in a 2 mL Eppendorf tubes, then 500 µL of cold water/methanol 70:30 *v*/*v* (−20 °C) containing 0.1% of perchloric acid (*v*/*v*) solvent were added. Samples were shaken with vortex for 20 min. Then, they were centrifuged using an Eppendorf Centrifuge 5427 R (Eppendorf, Hamburg, Germany) for 20 min 12,700 rpm at 4 °C. Supernatants were collected and introduced in a new 2 mL Eppendorf tubes. A second extraction was performed adding 500 µL of ddH_2_O + 0.1% perchloric acid (*v*/*v*) to leaves and roots, shaken for 5 min with vortex, and centrifuged for 20 min with 12,700 rpm at 4 °C. Supernatants were mixed and centrifuged for 10 min in order to eliminate suspended particles. Finally, supernatants were diluted three times with ddH_2_O + 0.1% formic acid (*v*/*v*) and introduced in 2 mL LC-MS vials. Metabolite analysis was achieved using an ultra-high-performance liquid chromatography (UPLC) Acquity H-Class system (Waters Corp, Milford, MA, USA), and high-resolution detection was performed by using a Xevo G2-S QToF mass spectrometer (Waters Corp, Milford, MA, USA) equipped with an electrospray ionization (ESI) source. A Phenomenex Luna® Omega PS C18 (100 × 2.1 mm, 1.6 µm) column (Torrance, CA, USA) was used to profile the organic acids. The mobile phase, comprising water containing 0.5% formic acid (A) and methanol: Water (70:30 *v*/*v*) containing 0.5% formic acid (B), was applied with the optimized gradient elution as follows: 100% A at 0–1 min, 100–20% A at 1–4 min, 20–0% A at 4–6.5 min, 0% A at 6.5–7.5 min, 0–100% A at 7.5–7.9 min, 100% A at 7.9–10 min. The flow rate was kept at 0.3 mL/min, column temperature was maintained at 35 °C. The injection volume for both columns was 10 µL and samples were maintained at 10 °C. The ESI source was used in negative ionization, source voltage was set to 2.5 kV and cone voltage was 30 V, whilst source temperature was maintained at 130 °C with a cone gas flow of 20 L/h. The desolvation temperature was at 500 °C, with a desolvation gas flow of 900 L/h. Leucine-Enkephalin was used as lockmass reference, (ion at m/z 556.2771 in positive mode), which was introduced by a lockspray at 10 µL min^−1^ for real-time data calibration. The MSE data were acquired in centroid mode using a scan range 50–800 Da, scan time 0.1 s, resolution was set at 20,000 full width half maximum (FWHM), and a collision energy ramp 40–80 V. GSH and GSSG were determined according to Ali et al., 2018.

Polyamine extraction was achieved using 20 mg of frozen ground leaves that were weighed in a 2 mL eppendorf tube (Eppendorf, Hamburg, Germany). Extraction was carried out by adding 1 mL of a solution of 70% H2O/29% MeOH/1.0% formic acid (*v*/*v*/*v*) at −20 °C using a Mixer Mill MM 400 (Retsch, Haan, Germany). Next, the tubes were stirred at room temperature for 30 min, and then centrifuged at 4 °C for 20 min (12,500 rpm), and the supernatant was transferred into new Eppendorf tubes. The supernatant was transferred to a LC/MS vial for analysis. Polyamines were analyzed by an ultra-high performance liquid chromatography coupled with tandem mass spectrometry (UHPLC–MS/MS) system. The separation and detection were achieved using a Nexera X2 UHPLC system (Shimadzu, Kyoto, Japan) coupled to a QTrap 6500+ mass spectrometer (Sciex, Concord, ON, Canada) equipped with an IonDriveTM turbo V electrospray (ESI) source.

### 4.6. RNA Extraction and Gene Expression Analysis

The root and leaf samples (100 mg) of sugar beet plants were ground to a fine powder in the presence of liquid nitrogen and total RNA was extracted using a Nucleospin® 8 RNA kit following the manufacturer’s protocol (Macherey-Nagel, Düren, Germany). The quality and yield of all RNA samples were analyzed and checked in a 4200 Tapestation (Agilent Technologies, Santa Clara, CA, USA), followed by DNase treatment and cDNA synthesis from 1 µg RNA using iScript^TM^ gDNA clear cDNA synthesis kit (Bio-Rad, Hercules, CA, USA). Quantitative RT-PCR (qPCR) analysis was performed in a total volume of 10 µL using Universal SYBR Green Supermix (Bio-Rad, Hercules, CA, USA) in Real-Time PCR Detection System (Bio-Rad, Hercules, CA, USA). The qPCR reactions were performed in technical triplicates using independent cDNA reactions for each biological replicate and 300 nM of gene-specific primer pairs. Specific primers for all candidate genes were designed using Primer3 software (version 0.4.0) and are listed in Supplementary Table S2. The thermal cycler protocol was 98 °C for 3 min, 40 cycles of 98 °C for 15 s, 60 °C for 30 s, 72 °C for 15 s, and a final 5-min extension at 72 °C. The expression of all candidate genes were normalized against three sugar beet reference genes, namely, *EF1α*, *18SrRNA*, and *β-tubulin*. All qPCR expression data were acquired and analyzed using CFX Maestro Software Version 1.0 (Bio-Rad, Hercules, CA, USA).

### 4.7. Statistical Analysis

Data are represented as mean ± standard deviation (SD) or standard error of the mean (SEM) for *n* = 6. The analysis of variance (ANOVA) and the post-hoc Student–Newman–Keuls (SNK) (R software) were employed to analyze the data and marked by different letters when significantly different (*p* < 0.05).

## 5. Conclusions

**Figure 8:**
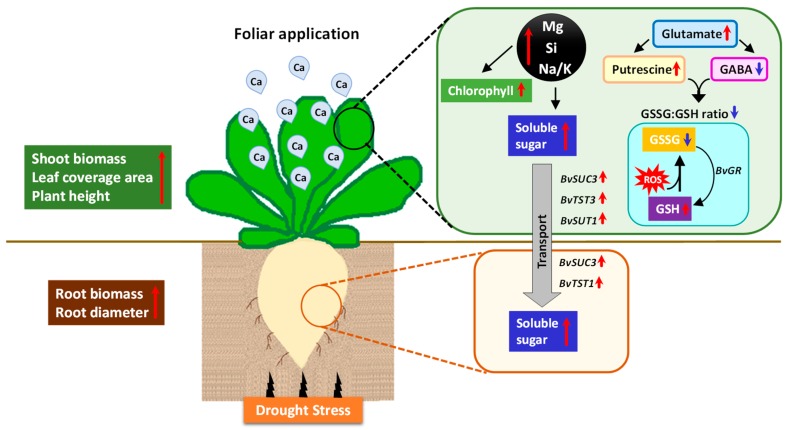
Schematic model representing the regulatory role of Ca^2+^ on the response of sugar beet plants to drought stress. Ca^2+^ treated sugar beet plants effectively tolerated drought stress by regulating the concentration of Mg and Si in the leaves which consequently enhanced sugar metabolism both at the metabolic and transcriptional level. Ca^2+^ also increased the Na/K ratio in leaf displaying the possible role of Na in osmotic adjustment under drought stress. Shoots displayed increased leaf coverage area, higher biomass, and higher chlorophyll, along with increase in the beetroot diameter and beetroot biomass. Additionally, the lower GSSG/GSH ratio, higher putrescine levels, and reduced GABA level clearly showed that the Ca^2+^ treated plants were able to efficiently tolerate drought-induced oxidative stress. (Red arrows = increase/upregulation, blue arrows = decrease/downregulation).

In our study, we show that the foliar application of Ca^2+^ to sugar beet plants exposed to drought stress regulates plant mineral status (Mg and Si), sugar metabolism both at metabolic and transcriptional levels, influences plant metabolism and balanced redox state and thus increased drought stress tolerance. In leaves, foliar application of Ca^2+^ increased plant dry matter as well as chlorophyll levels under drought. Also, the application of Ca^2+^ enhanced the concentrations of Mg and Si which could positively impact the sugar synthesis and transcriptionally regulate the genes involved in sugar transport (*BvSUC3* and *BvTST3*). Additionally, plants showed a strong tolerance to drought induced-oxidative stress by regulating glutathione pools (lower GSSG/GSH ratio) which was further supported by a higher putrescine level and lower GABA concentrations. In beetroot, the remarkable effect of Ca^2+^ was higher Suc storage under drought stress. Altogether, our findings demonstrate that foliar application of Ca^2+^ enabled drought-stressed sugar beet plants to cope better with stress by maintaining efficient shoot growth and allocating relatively more Suc to the beetroots (Figure 8).

## Figures and Tables

**Table 1:** Influence of foliar application of Ca^2+^ on the beetroot and leaf elemental composition in sugar beet plants exposed to drought stress. Plants were grown in pots for a duration of 8 weeks. Five-week-old sugar beet plants were kept at 90% field capacity as control or exposed to drought stress (30% field capacity) for a duration of 3 weeks. Ca^2+^ was applied at BBCH14 and BBCH18 in concentration of 5 L ha^−1^ corresponding to 540 g Ca ha^−1^. Leaves and beetroot were harvested at 60 days after sowing for elemental analysis. Bars indicate means ± SEM. Different letters denote significant differences according to ANOVA followed by SNK test (*p* < 0.05; *n* = 6) and ns denotes non-significant differences. Abbreviations are: N, nitrogen; K, potassium; P, phosphorus; Mg, magnesium; Ca, calcium; S, sulfur; Na, sodium; Mn, manganese; Zn, zinc; Fe, iron; B, boron; Cu, copper; and Si, silicon.

		Beetroot (mg g^−1^ DW)			Leaves (mg g^−1^ DW)		
		Control	Drought	Drought + Ca	Control	Drought	Drought + Ca
Macro-elements	N	8.45 ± 0.60 ^b^	9.07 ± 0.75 ^ab^	9.53 ± 0.19 ^a^	28.05 ± 4.98 ^ns^	26.22 ± 7.25 ^ns^	30.16 ± 4.03 ^ns^
	K	19.07 ± 2.17 ^a^	18.22 ± 2.69 ^a^	13.00 ± 1.22 ^b^	51.71 ± 16.71 ^ns^	54.10 ± 15.05 ^ns^	39.03 ± 3.16 ^ns^
	P	4.21 ± 0.46 ^a^	4.20 ± 0.65 ^a^	3.01 ± 0.08 ^b^	17.24 ± 6.96 ^a^	15.51 ± 5.33 ^a^	8.11 ± 1.41 ^b^
	Mg	1.70 ± 0.30 ^ns^	1.78 ± 0.19 ^ns^	1.68 ± 0.06 ^ns^	9.30 ± 0.70 ^b^	10.22 ± 2.09 ^b^	14.61 ± 1.99 ^a^
	Ca	1.46 ± 0.10 ^ns^	1.49 ± 0.04 ^ns^	1.37 ± 0.09 ^ns^	12.22 ± 2.62 ^ns^	11.35 ± 2.08 ^ns^	13.11 ± 1.24 ^ns^
	S	0.67 ± 0.04 ^ns^	0.71 ± 0.06 ^ns^	0.68 ± 0.08 ^ns^	5.64 ± 1.11 ^ns^	5.15 ± 0.90 ^ns^	4.61 ± 0.19 ^ns^
	Na	0.61 ± 0.25 ^ns^	0.38 ± 0.10 ^ns^	0.40 ± 0.04 ^ns^	11.31 ± 3.14 ^ns^	11.86 ± 3.54 ^ns^	15.29 ± 1.46 ^ns^
Micro-elements	Mn	0.051 ± 0.035 ^ab^	0.083 ± 0.053 ^a^	0.012 ± 0.002 ^b^	0.454 ± 0.331 ^a^	0.543 ± 0.401 ^a^	0.051 ± 0.005 ^b^
	Zn	0.038 ± 0.007 ^a^	0.043 ± 0.007 ^a^	0.030 ± 0.003 ^b^	0.313 ± 0.190 ^a^	0.167 ± 0.045 ^ab^	0.113 ± 0.004 ^b^
	Fe	0.017 ± 0.002 ^b^	0.025 ± 0.007 ^a^	0.016 ± 0.002 ^b^	0.070 ± 0.007 ^ns^	0.070 ± 0.005 ^ns^	0.073 ± 0.006 ^ns^
	B	0.014 ± 0.001 ^ns^	0.015 ± 0.001 ^ns^	0.013 ± 0.002 ^ns^	0.058 ± 0.014 ^ns^	0.060 ± 0.014 ^ns^	0.065 ± 0.006 ^ns^
	Cu	0.007 ± 0.001 ^a^	0.007 ± 0.001 ^a^	0.005 ± 0.0004 ^b^	0.019 ± 0.005 ^a^	0.013 ± 0.003 ^ab^	0.009 ± 0.001 ^b^
Beneficial	Si	0.010 ± 0.003 ^ns^	0.009 ± 0.002 ^ns^	0.009 ± 0.002 ^ns^	0.230 ± 0.104 ^b^	0.221 ± 0.084 ^b^	0.338 ± 0.053 ^a^
Ratio	Na/K	0.029 ± 0.014 ^ns^	0.021 ± 0.004 ^ns^	0.03 ± 0.003 ^ns^	0.25 ± 0.14 ^ns^	0.27 ± 0.11 ^ns^	0.39 ± 0.035 ^ns^

## Supplementary Materials

Supplementary materials can be found at https://www.mdpi.com/1422-0067/20/15/3777/s1.

## Abbreviations

DAA	Day after application
Glu	Glucose
Fru	Fructose
Suc	Sucrose
GABA	Gamma-aminobutyric acid
Put	Putrescine
GSH	Reduced glutathione
GSSG	Oxidized Glutathione
*BvCMO*	Choline monooxygenase
*BvDREB2A*	Dehydration responsive element-binding 2A
