# Supplementary material for: Calcium Application Enhances Drought Stress Tolerance in Sugar Beet and Promotes Plant Biomass and Beetroot Sucrose Concentration
Source: Int J Mol Sci. 2019 Aug 2;20(15):3777. doi: 10.3390/ijms20153777 (PMC6696248; doi:10.3390/ijms20153777)
Supplement: Supplementary file 1 [file ijms-20-03777-s001.zip › ijms-542404-SI.docx]

**Supplemental Table S1: Influence of foliar application of Ca on amino acid and organic acid concentrations of both roots and leaves in sugar beet plants exposed to drought stress.** Plants were grown in pots for a duration of eight weeks. Five-week-old sugar beet plants were kept at 90% field capacity as control or exposed to drought stress (30% field capacity) and for a duration of three weeks. Ca was applied at BBCH14 and BBCH18 in concentration of 5L ha^-1^ corresponding to 540g Ca ha^-1^. Leaves and beetroot were harvested at 60 days after sowing for analysis of amino and organic acids. Bars indicate means ± SEM. Different letters denote significant differences according to ANOVA followed by SNK test (p < 0.05; n = 6). Abbreviations are: Ala, alanine; Asn, asparagine; Asp, aspartate; GABA, gamma-aminobutyric acid; Gln, glutamine; Glu, glutamate; Gly, glycine; His, histidine; Ile, isoleucine; Leu, leucine; Lys, lysine; Pro, proline; Ser, serine; Thr, threonine; Trp, tryptophan; Tyr, tyrosine; Val, valine; and NA, not detected.

|  |  | **Beetroot** | | | **Leaves** | | |
| --- | --- | --- | --- | --- | --- | --- | --- |
|  |  | **Control** | **Drought** | **Drought+ Ca** | **Control** | **Drought** | **Drought+ Ca** |
| **Amino acids (mg g^-1^ DW)** | **Ala** | 0.09 ± 0.06^ns^ | 0.09 ± 0.04^ns^ | 0.07 ± 0.01^ns^ | 0.19 ± 0.03^ns^ | 0.19 ± 0.08^ns^ | 0.21 ± 0.03^ns^ |
|  | **Asn** | 0.16 ± 0.03^b^ | 0.17 ± 0.02^b^ | 0.24 ± 0.04^a^ | 0.19 ± 0.03^ns^ | 0.18 ± 0.08^ns^ | 0.19 ± 0.03^ns^ |
|  | **Asp** | 0.07 ± 0.01^ns^ | 0.07 ± 0.01^ns^ | 0.08 ± 0.01^ns^ | NA | NA | 0.04 ± 0.02 |
|  | **GABA** | 0.47 ± 0.11^b^ | 0.81 ± 0.09^a^ | 0.89 ± 0.20^a^ | 0.67 ± 0.13^b^ | 1.04 ± 0.36^a^ | 0.64 ± 0.09^b^ |
|  | **Gln** | 1.29 ± 0.11^b^ | 1.41 ± 0.35^b^ | 1.82 ± 0.32^a^ | 0.29 ± 0.06^a^ | 0.19 ± 0.06^b^ | 0.25 ± 0.05^ab^ |
|  | **Glu** | 0.83 ± 0.08^a^ | 0.51 ± 0.18^b^ | 0.34 ± 0.21^b^ | 1.88 ± 0.10^a^ | 1.40 ± 0.19^b^ | 1.98 ± 0.21^a^ |
|  | **Gly** | 0.02 ± 0.01^b^ | 0.02 ± 0.01^b^ | 0.03 ± 0.01^a^ | NA | NA | NA |
|  | **His** | 0.03 ± 0.002^ns^ | 0.04 ± 0.006^ns^ | 0.04 ± 0.004^ns^ | 0.07 ± 0.03^b^ | 0.13 ± 0.04^a^ | 0.08 ± 0.01^b^ |
|  | **Ile** | 0.11 ± 0.05^b^ | 0.15 ± 0.04^ab^ | 0.19 ± 0.04^a^ | 0.14 ± 0.03^ns^ | 0.12 ± 0.01^ns^ | 0.13 ± 0.02^ns^ |
|  | **Leu** | 0.06 ± 0.02^b^ | 0.10 ± 0.03^ab^ | 0.14 ± 0.04^a^ | NA | NA | NA |
|  | **Lys** | 0.06 ± 0.04^b^ | 0.06 ± 0.03^b^ | 0.13 ± 0.06^a^ | 0.02 ± 0.002^ns^ | NA | 0.03 ± 0.01^ns^ |
|  | **Pro** | 0.03 ± 0.004^ns^ | 0.03 ± 0.002^ns^ | 0.04 ± 0.02^ns^ | 0.06 ± 0.01^ns^ | 0.05 ± 0.01^ns^ | 0.05 ± 0.01^ns^ |
|  | **Ser** | 0.15 ± 0.003^b^ | 0.20 ± 0.03^a^ | 0.21 ± 0.03^a^ | 0.16 ± 0.02^ns^ | 0.14 ± 0.04^ns^ | 0.22 ± 0.08^ns^ |
|  | **Thr** | NA | NA | NA | 0.09 ± 0.02^ns^ | 0.09 ± 0.02^ns^ | 0.11 ± 0.01^ns^ |
|  | **Trp** | 0.05 ± 0.02^b^ | 0.09 ± 0.02^a^ | 0.09 ± 0.02^a^ | 0.06 ± 0.03^b^ | 0.11 ± 0.02^a^ | 0.08 ± 0.01^b^ |
|  | **Tyr** | NA | NA | NA | 0.11 ± 0.02^ns^ | 0.09 ± 0.02^ns^ | 0.09 ± 0.02^ns^ |
|  | **Val** | 0.06 ± 0.02^ns^ | 0.06 ± 0.01^ns^ | 0.08 ± 0.03^ns^ | 0.08 ± 0.02^ns^ | 0.06 ± 0.01^ns^ | 0.08 ± 0.01^ns^ |
| **Organic acids (µg g^-1^ FW)** | **Citrate** | 99.54 ± 6.05^ab^ | 113.12 ± 21.56^a^ | 84.28 ± 7.61^b^ | 228.69 ± 47.42^ns^ | 265.39 ± 65.60^ns^ | 249.95 ± 41.68^ns^ |
|  | **Fumarate** | 21.39 ± 2.29^ns^ | 23.25 ± 2.74^ns^ | 22.51 ± 3.06^ns^ | 327.87 ± 80.33^b^ | 563.10 ± 227.32^ab^ | 680.03 ± 209.92^a^ |
|  | **Isocitrate** | 21.88 ± 5.26^ns^ | 27.24 ± 4.07^ns^ | 23.34 ± 3.78^ns^ | 42.28 ± 8.05^b^ | 51.99 ± 15.85^b^ | 75.03 ± 19.90^a^ |
|  | **Malate** | 22.23 ± 3.15^ns^ | 24.02 ± 3.94^ns^ | 26.70 ± 3.76^ns^ | 193.19 ± 33.84^b^ | 282.18 ± 81.81^a^ | 306.86 ± 40.96^a^ |
|  | **Succinate** | 3.33 ± 0.28^ns^ | 2.65 ± 0.48^ns^ | 2.60 ± 0.70^ns^ | 4.02 ± 0.54^ns^ | 3.52 ± 0.68^ns^ | 3.80 ± 0.55^ns^ |

**Supplemental Table S2.** List of primers used for qRT-PCR.

| **GENE** | **FORWARD PRIMER** | **REVERSE PRIMER** | **AMPLICON SIZE (BP)** |
| --- | --- | --- | --- |
| *BvSUC3* | TTGACACTGACTGGATGGGA | AGCACCTTCTCTGACACCTT | 98 |
| *BvTST1* | TCACTTACTCCTTGCCCGTC | GAGATGACACAAACCACAGCA | 82 |
| *BvTST3* | AGGGCTTGGAACTGGGAAA | TATCTGTGGTGGCTTCTTCATC | 80 |
| *BvSUT1* | CCTCAGATGTTTGTGTCGGTAA | AAAGGAAAGGATGGCACTGG | 117 |
| *BvGR* | CGCCCAAGGACAACTCTG | TCAGCCTCTTCGTGTTAGGA | 133 |
| *BvCMO* | TGAGCCTGCCTTTTATTCCC | AAGTTCGCCTTGACCATCTC | 157 |
| *BvDREB2A* | ATCGGTGGCTGAAACTCTTG | CCTAACAGGCTTACCTTCCTTG | 82 |
| *BvEF1α* | ATTGCCACACCTCCCACA | ACCATACCAGCATCACCATTC | 121 |
| *Bv18srRNA* | AACCATAAACGATGCCGACC | TTTCAGCCTTGCGACCATAC | 115 |
| *Bv*β*-tubulin* | CACCAAAGAAGTAGATGAGCAGA | GTTACCAACGAATGTAGAAGCCA | 145 |

**Supplemental Figure S1: Influence of foliar application of Ca on the expression levels of drought stress markers in sugar beet exposed to drought stress.** (A) Relative *BvCMO* mRNA levels in leaves and (B) relative *BvDREB2A* mRNA levels in leaves. Plants were grown in pots for a duration of eight weeks. Five-week-old sugar beet plants were kept at 90% field capacity as control or exposed to drought stress (30% field capacity) and for a duration of three weeks. Ca was applied at BBCH14 and BBCH18 in concentration of 5L ha^-1^ corresponding to 540g Ca ha^-1^. Leaves were harvested at 60 days after sowing for gene expression analysis. Bars indicate means ± SD (n = 6).

**Supplemental Figure S2: Influence of foliar application of Ca on total solute concentrations in sugar beet plants exposed to drought stress.** (A) Total solutes (amino acids, Na and K) without sucrose; (B) Total solutes (amino acids, Na, K) with sucrose. Plants were grown in pots for a duration of eight weeks. Five-week-old sugar beet plants were kept at 90% field capacity as control or exposed to drought stress (30% field capacity) and for a duration of three weeks. Ca was applied at BBCH14 and BBCH18 in concentration of 5L ha^-1^ corresponding to 540g Ca ha^-1^. Leaves and beetroot were harvested at 60 days after sowing for total solute and sugar analysis. Bars indicate means ± SD. Different letters denote significant differences according to ANOVA followed by SNK test (p < 0.05; n = 6).
